# Supplementary material for: Cloud BioLinux: pre-configured and on-demand bioinformatics computing for the genomics community
Source: BMC Bioinformatics. 2012 Mar 19;13:42. doi: 10.1186/1471-2105-13-42 (PMC3372431; doi:10.1186/1471-2105-13-42)
Supplement: Additional file 1 — Supplementary 1 Cloud BioLinux software documentation in the form of a mini, self-contained website. Users need to download and uncompress the .zip file, and open through a web browser the "index.html" file available on the main directory. (ZIP 1823 kb). [file 1471-2105-13-42-S1.ZIP › Cloud-BioLinux-Package-Documentation/Utils.html]

Bio-Linux Software Documentation Pages

Back to search form

### Utils

|  |  |
| --- | --- |
| Category | Applications |
| Utils | biojava    extract\_from\_pdb    bioperl |
| SubCategory | Applications |
| Assembly reformatting | assembly-conversion-tools    caf2gap    caftools    gap2caf    mira-3rd-party |
| Database\_creation | aaindexextract    cutgextract    jaspextract    printsextract    prosextract    rebaseextract    tfextract |
| Database\_indexing | dbiblast    dbifasta    dbiflat    dbigcg    dbxfasta    dbxflat    dbxgcg    dbxstat |
| Misc | cytoscape    dendroscope    dolmove    drawgram    drawtree    embossdata    embossversion    entrails    Lamarc    mesquite    move    njplot |
| Sequence\_cleaning | chimeraslayer    denoiser    lucy    qiime |
| Sequence\_info | extract\_from\_pdb    FastQC    seqstat |
| Sequence\_reformatting | 2ft    clustalw    clustalx    copymat    extract\_from\_pdb    gtop    hmmconvert    htop    makemat    pfmake    psa2msa    ptof    ptoh    readseq    sfetch    sreformat |
| Software\_development | bioconductor    biojava    bioperl    bioruby |
